# Nuclear Prospero allows one-division potential to neural precursors and post-mitotic status to neurons via opposite regulation of Cyclin E

**DOI:** 10.1371/journal.pgen.1010339

**Published:** 2022-08-08

**Authors:** Jordan Mar, Kalpana Makhijani, Denise Flaherty, Krishna Moorthi Bhat

**Affiliations:** Department of Molecular Medicine, Morsani College of Medicine, University of South Florida, Tampa, Florida, United States of America; New York University, UNITED STATES

## Abstract

In Drosophila embryonic CNS, the multipotential stem cells called neuroblasts (NBs) divide by self-renewing asymmetric division and generate bipotential precursors called ganglion mother cells (GMCs). GMCs divide only once to generate two distinct post-mitotic neurons. The genes and the pathways that confer a single division potential to precursor cells or how neurons become post-mitotic are unknown. It has been suggested that the homeodomain protein Prospero (Pros) when localized to the nucleus, limits the stem-cell potential of precursors. Here we show that nuclear Prospero is phosphorylated, where it binds to chromatin. In NB lineages such as MP2, or GMC lineages such as GMC4-2a, Pros allows the one-division potential, as well as the post-mitotic status of progeny neurons. These events are mediated by augmenting the expression of Cyclin E in the precursor and repressing the expression in post-mitotic neurons. Thus, in the absence of Pros, Cyclin E is downregulated in the MP2 cell. Consequently, MP2 fails to divide, instead, it differentiates into one of the two progeny neurons. In progeny cells, Pros reverses its role and augments the downregulation of *Cyclin E*, allowing neurons to exit the cell cycle. Thus, in older *pros* mutant embryos Cyclin E is upregulated in progeny cells. These results elucidate a long-standing problem of division potential of precursors and post-mitotic status of progeny cells and how fine-tuning *cyclin E expression* in the opposite direction controls these fundamental cellular events. This work also sheds light on the post-translational modification of Pros that determines its cytoplasmic versus nuclear localization.

## Author summary

In this study, first, we provide insight into the role of phosphorylation in the cytoplasmic versus nuclear localization of Prospero. It is non-phosphorylated and cytoplasmic in self-renewing multipotential precursor stem cells, whereas phosphorylated and nuclear in bipotential precursor cells in the CNS. Second, we show that nuclear translocation and binding of Pros to chromatin specifies the single-division potential of bipotential precursor cells. Our experiments further show that this is achieved by augmenting the upregulation of Cyclin E expression. However, following the division, in the progeny cells, Pros augments the downregulation of Cyclin E expression, allowing the progeny cells to exit the cell cycle and become post-mitotic. These results shed light on long-standing problems of the single division potential of precursors and the post-mitotic status of progeny cells. Prospero via regulating Cyclin E in opposite directions at different developmental time points appears to achieve these critical milestones during neurogenesis.

## Introduction

It has been well-established that the primary precursor NBs divide many times in the fly embryonic nerve cord, whereas the progeny secondary precursor GMCs divide only once [1–4]. Most of the studies over the years have focused on asymmetric fate specification by asymmetrically localized determinants [2–13], or self-renewing asymmetric division of NBs [14–16] or in some cases GMCs [17]. But we do not know how precursors such as MP2 or GMCs acquire the potential to divide only once, or how the progeny cells exit the cell cycle and become post-mitotic. Subcellular localization of determinants and protein modifications are considered the prime targets for mediating these processes. For example, in the developing Drosophila embryonic ventral nerve cord (VNC), there are proteins such as Notch, Numb, and Inscuteable (Insc), that are asymmetrically localized within a precursor. Our previous work showed that Notch signaling polarizes Insc to the apical pole, which in turn segregates Numb to the basal pole of a GMC [9]. Notch and Numb have an antagonistic role: wherever the Numb protein is concentrated or localized, it will block Notch-signaling in that region. These interactions in the precursor cells begin the process and culminate in the specification of different cell fates to progeny cells. There are also proteins such as Insc, Neuralized, or Numb that are asymmetrically localized in NBs and then segregate to the progeny GMCs during the self-renewing asymmetric division of NBs [10,13,18,19,20,21]. They maintain their cytoplasmic asymmetric distribution in GMCs and provide a mechanism to promote terminal asymmetric division of such cells. These proteins may also be involved in the fate specification of progeny neurons as they segregate to one cell or the other during GMC division [22,23].

Then there is another category of proteins, such as Prospero (Pros). Pros is a homeodomain protein [22,23] and it is asymmetrically localized in the cortex of NBs [5–8]. When an NB divides, Pros translocates into the nucleus of GMCs. It is not clear what is the role of asymmetrically localized cytoplasmic Pros in NBs or nuclear-localized Pros in GMCs and in NBs such as MP2, a bipotential precursor [1,24]. One idea is that cytoplasmic Pros promotes NBs to undergo self-renewing asymmetric division, and nuclear Pros prevents GMCs and MP2 from undergoing stem cell type of division. As a default, cells become post-mitotic. The suggestion that nuclear Pros is a stem-cell division inhibitor and cytoplasmic Pros is a stem cell promoter is based on the previous reports that in loss of function for *pros*, or in vitro culture of cells from *pros* embryonic CNS, precursor cells de-differentiate and become stem cells [25,26]. It was further suggested that cytoplasmic versus nuclear localizations between NBs and GMCs allow the expression of stem cell factors in self-renewing NBs, and repression of these genes in GMCs [25].

The above mechanistic hypothesis did not account for the programming of GMCs or an MP2 to divide only once, nor did it address the possibility that Pros in bipotential precursors confers a single division potential to them. Based on our preliminary work on Pros, we wondered if, at some point in the evolutionary past, GMCs might not have been programmed to divide at all but directly differentiate into neurons. Credence to this possibility comes from the fact that if GMCs are blocked from dividing using cell cycle mutants such as *cyclin A*, *regulator of cyclin A* or *string*, they directly differentiate into one of the two progeny neurons [27,28]. During evolution, perhaps due to pressure to increase the number of neurons in the developing nervous system, GMCs might have acquired the ability to divide once. We entertained the possibility that nuclear Pros may be involved in inducing a GMC or MP2, where Pros is nuclear, to divide once. The cytoplasmic localization in NBs may be a mechanism to segregate Pros to GMCs.

The Pros protein is also a phosphoprotein [29]. Subcellular and differential localization of proteins is often mediated by post-translational modifications such as glycosylation [30,31]. While glycosylation plays a key role in protein secretion and so on [30,32,33], phosphorylation is directly related to the activity of a protein [34]. Phosphorylation, which mainly occurs at the side chains of serine, threonine, and tyrosine, is associated with the activity of a large subset of proteins and is also a key to regulating protein function. For example, a protein could be changed from an inactive state to an active state or vice versa [35,36] via conformational changes. These changes can regulate the catalytic activity of a phosphoprotein, thus, can either activate or inactivate them. Phosphorylation can also recruit neighboring proteins that recognize and bind phosphomotifs [37,38]. Since Pros is a phosphoprotein [29], we sought to re-examine the nuclear versus cytoplasmic localization and the phosphorylation status of Pros.

In this study, we used MP2, which behaves like a GMC by dividing only once to generate two post-mitotic neurons [1,24]. We argue that it is an NB since it is formed during the first wave of NB formation (as an S1 NB) and under the control of proneural and neurogenic genes. We also examined a well-studied GMC lineage (GMC4-2a), which generates two post-mitotic neurons, RP2 and sib. Additionally, we also examined NB7-3, a typical NB lineage, if it generates extra neurons in *pros* mutants. Using these lineages, we show that Pros specifies a single division potential to MP2 and GMC4-2a and post-mitotic status to progeny neurons. Our results also show that NB7-3 does not generate extra neurons if anything, fewer cells. The single division potential and post-mitotic status are conferred in part via regulating Cyclin E levels in opposite directions in precursors versus post-mitotic cells. However, it is not clear if this mechanism extends to all GMCs or bipotential precursors and neurons. We also show that the cytoplasmic to nuclear localization of Pros correlates with its phosphorylation status. It appears that phosphorylation allows Pros to become nuclear and bind to chromatin. These findings provide a better understanding of the function of Pros during lineage elaboration, the molecular biology of the division potential of precursors, and the achievement of the post-mitotic status of progeny neurons. These are long-standing problems in neurobiology.

## Results

### Phosphorylated Prospero is nuclear in neural precursor cells

**Fig 1:**
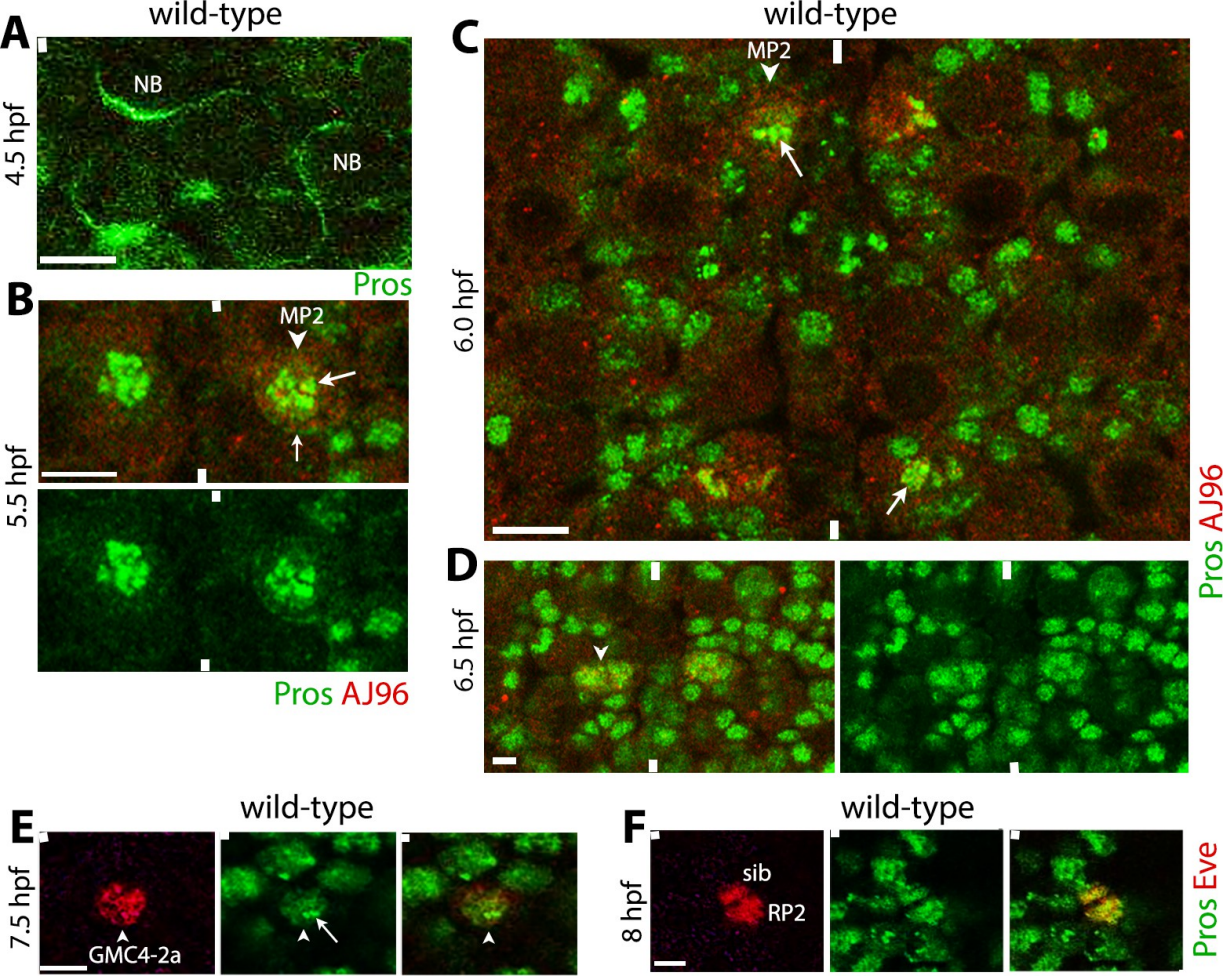
The Pros protein binds to chromatin in MP2 and GMCs in the embryonic ventral nerve cord. Embryos are stained for Pros (A), double-stained for Pros and AJ96 (B-D) or Pros and Eve (E, F). Pros is in Green, AJ96 or Eve are in Red. The anterior end is up, the midline is marked by vertical lines. The scale bar is 10 μm (A-C), 5 μm (D, E), and 3 μm (F). (**A**): Pros is cytoplasmic and asymmetrically localized at the cortex in NBs. (**B, C**): In MP2 (arrowhead), Pros is nuclear and bound to chromatin in MP2 (arrow). Residual cytoplasmic Pros is also seen in MP2 (small arrow). (**D):** Pros is also nuclear and bound to chromatin in newly formed progeny of MP2 (arrowhead). (**E, F**): Pros localizes to the chromatin in GMCs. Shown is an Eve-positive GMC4-2a (E, arrowhead); the chromatin-bound Pros is marked by an arrow. Pros is nuclear in RP2 and sib cells (F), the two daughter cells of GMC4-2a.

Immunohistochemistry of the ventral nerve cord of Drosophila embryo with anti-Pros shows that while Pros is asymmetric in the cytoplasm of NBs (Fig 1A), it is localized to the nucleus, and specifically to the chromatin within the nucleus in MP2 and GMCs (Fig 1B–1C and Fig 1E). The chromatin localization in MP2 appears to be extensive, whereas in GMCs such as GMC4-2a, it may be more restricted (compare Fig 1B–1E). However, there are GMCs with extensive localization versus GMCs with more restricted ones, which may underlie different cell fate potentials since Pros is also involved in the specification of cell identity [22,23]. That Pros is chromatin-bound is also indicated by previous studies [25,39]. Additionally, staining of GMCs and other tissues with an antibody against acetylated Histone 4 (H4K16Ac), a well-known chromatin protein that specifically binds to X-chromosome in males and facilitates recruitment of the MSL-complex [40,41] shows a similar staining pattern, but restricted to X-chromosome in males (S1 Fig) [42]. One can also realize that the extent of chromatin-bound by Pros is extensive and likely covers all chromosomes compared to the H4K16Ac binding (Fig 1B and 1E versus S1 Fig), where it can regulate a large number of genes.

**Fig 2:**
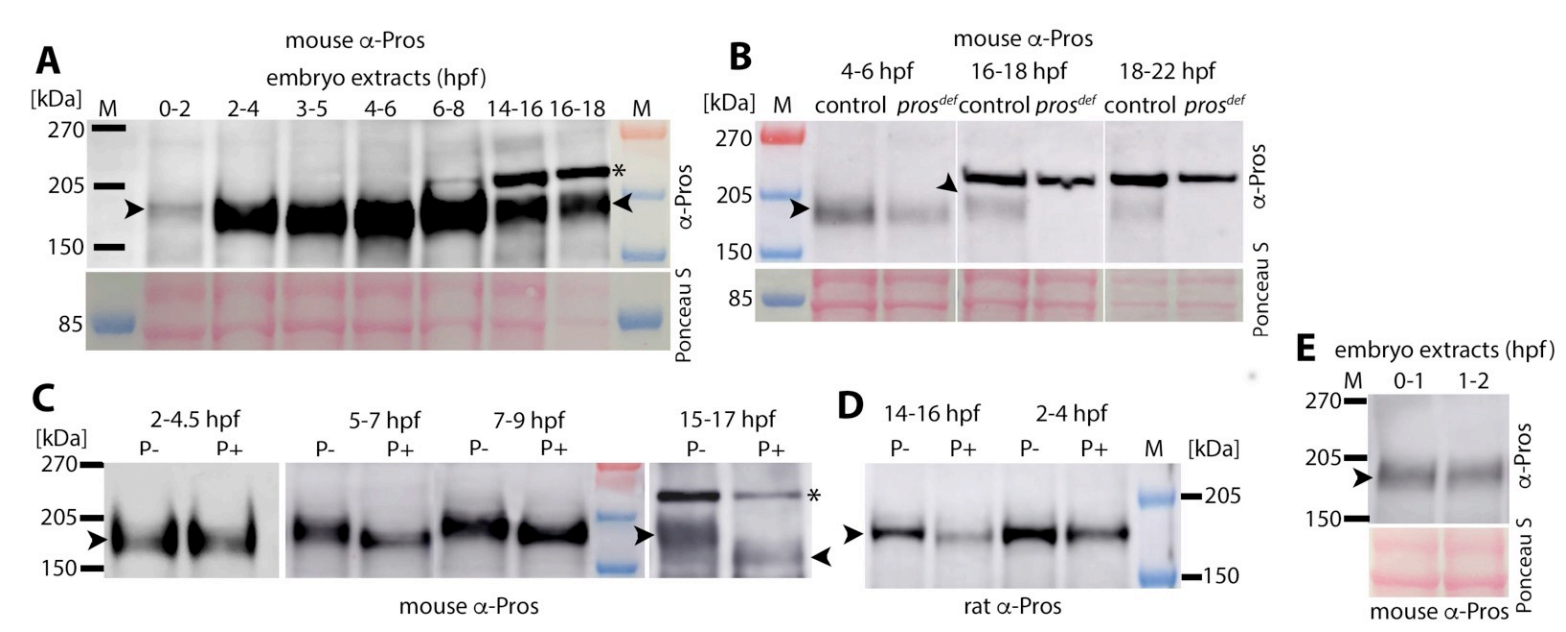
The phosphorylation of Pros correlates with its nuclear localization. Western blotting analysis of embryo extracts with anti-Pros antibody. Ponceau staining of the membrane is to determine the loading control. They are from the lower molecular mass region and may or may not perfectly align with the higher molecular mass region depending on the way the gel is run. The arrowhead marks the Pros band (both non-phosphorylated and phosphorylated). The star marks the background band, which is present in older stage embryos. **(A)**: Developmental Western profile of Pros. The nerve cord in 2–5 hpf embryos have only NBs, 4–6 hpf embryos have mostly NBs and few GMCs. The Pros protein migrates at 170–180 kDa in extracts from these embryos. Embryos older than 8 hpf have a significant number of GMCs and neurons; by 14 hpf of age (or older), the nerve cord has mostly neurons. The molecular mass of Pros in embryos older than 6 hpf shifts to 180–190 kDa. The data come from the same gel-blot. **(B)**: Both the lower and the higher molecular mass Pros band is absent in extracts from older stage embryos (16–22 hpf) homozygous for a deficiency that removes the *pros* gene (lanes 4 and 6). A reduced level of Pros is present in 4–6 hpf homozygous deficiency embryos (lane 2), which is likely from the maternal deposition of Pros (see panel E). The upper non-specific protein band (indicated by a star) is present in *pros* deficiency embryos. The data come from the same gel blot. **(C)**: Western blotting analysis of embryo extracts from different developmental time points treated with a phosphatase enzyme. The arrowhead marks the Pros band. The lower molecular mass Pros band in 2–4.5 hpf extracts (when the VNC has only NBs with cytoplasmic Pros) is unaffected by the phosphatase treatment, indicating that it is non-phosphorylated. The higher molecular mass Pros gets reduced to the lower molecular mass Pros with the phosphatase treatment of extracts from older stage embryos, indicating Pros is phosphorylated in older stage embryos (when the VNC has mostly GMCs and neurons with nuclear Pros). The non-specific higher molecular weight band (indicated by a star) is phosphatase resistant (the last lane), while the higher molecular mass phosphorylated Pros is reduced to the lower molecular mass non-phosphorylated Pros with the phosphatase treatment (compare the last two lanes). The data come from three different gel blots. **(D):** Western-blotting analysis using an anti-Pros antibody raised in rat. The Pros band in extracts from 2–4 hpf old embryos is not phosphorylated, whereas it is phosphorylated in extracts from 14–16 hpf embryos, and sensitive to phosphatase treatment. This antibody does not recognize the non-specific band. The data come from the same gel blot. **(E):** The maternally deposited Pros is of the lower molecular mass (non-phosphorylated); its level is higher in 0–1 hpf embryos compared to 1–2 hpf embryos, indicative of a maternal deposition. The maternally deposited Pros appears to last up to 6–7 hpf of age (see text). The data come from the same gel blot.

Pros is a phosphoprotein [29]. Because of the subcellular localization dynamics of Pros during neurogenesis, we examined its phosphorylation status to cytoplasmic versus chromatin localization by performing developmental Westerns and dephosphorylation experiments (Fig 2). We found that embryo extracts from stages that have only NBs (<5 hpf) have faster-migrating Pros band of 170–180 kDa molecular mass (Fig 2A and 2B, arrowhead). But extracts from later stages, when a significant number of GMCs with their nuclear Pros are present (>6 hpf), have a higher molecular mass Pros band of 180–190 kDa (Fig 2A and 2B, arrowhead). Neither the fast nor the slow migrating Pros bands were present in older stage *pros* deficiency embryos (Fig 2B). However, the lower molecular mass Pros band was present in 4–6 hpf *pros* deficiency homozygous embryos, indicating that the *pros* gene product(s) is likely maternally deposited (see also Fig 2E). We do not know how far into the development this deposition lasts, but the VNC in 6.5–7 hpf *pros* mutant embryos exhibit a partially penetrant neuronal identity specification defects [22], timing the exhaustion of maternal deposition to around 6.5–7 hpf. The partial penetrance of the defects in the mutant and the variability of these defects from embryo to embryo (see below) could be due to the maternal deposition and its depletion dynamics within and between embryos.

We next performed phosphatase treatment of embryo extracts to determine if the higher molecular mass Pros is phosphorylated. The phosphatase treatment of extracts from earlier stages did not alter the migration pattern/molecular mass of the Pros band (Fig 2C and 2D), however the treatment of extracts from later stages of development collapsed the higher molecular mass band into the same lower molecular mass band as the Pros from earlier stages of development (Fig 2C and 2D). These results indicate that the faster-migrating Pros band from earlier stages of development when the nerve cord has mostly NBs with cytoplasmic Pros is non-phosphorylated, whereas the slower-migrating Pros in later stages of development when Pros is almost exclusively nuclear/chromatin-bound is phosphorylated.

A previous study [29] suggested that nuclear Pros is non-phosphorylated, and the cytoplasmic Pros is phosphorylated. It is possible that a non-specific higher molecular mass band present in later stages of neurogenesis (Fig 2A, 2B and 2C, indicated by a star), with a robust presence seen only in embryos older than 13 hours of development, was misinterpreted as Pros band. This band was present in a deficiency that removed the *pros* gene (Fig 2B, last lane), therefore, we believe that it is a non-specific band. This band was also not sensitive to phosphatase treatment (Fig 2C, the last lane, indicated by a star). Moreover, this non-specific band was not recognized by another anti-Pros antibody raised in another species although it recognizes both the phosphorylated and the non-phosphorylated Pros (Fig 2D).

### MP2 lineage has fewer cells in *pros* mutant embryos

While the asymmetric localization of Pros in an NB stem cell (Fig 1A) facilitates its segregation to a GMC during NB division, it was hypothesized that the subsequent nuclear localization of Pros in GMCs represses their stem cell potential [25]. The idea is that Pros binds to promoter/enhancer sequences of genes that are required for a stem cell division (self-renewing asymmetric division) and represses their expression [25]. Thus, GMCs could not undergo a stem cell-division, whereas in *pros* mutants, GMCs would adopt the parental identity and undergo multiple divisions. We examined the MP2 lineage in *pros* loss of function mutants using the marker Odd-skipped (Odd). Odd is expressed in MP2 as well as its progeny dMP2 but not in its sibling vMP2 [24].

**Fig 3:**
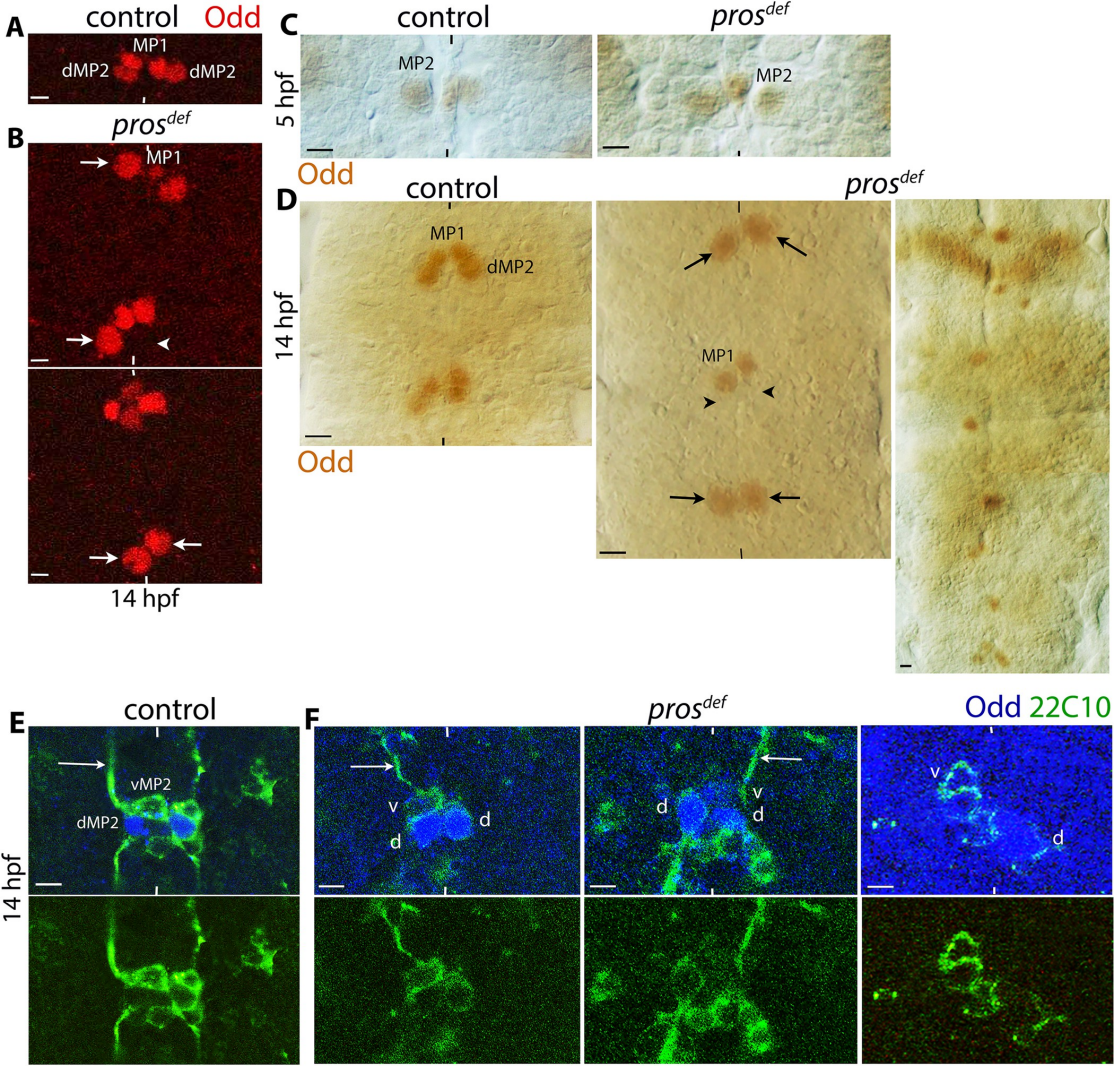
Fewer and not more cells are observed in the MP2 lineage in *pros* mutant embryos. Embryos are stained for Odd (A-D) and double-stained for Odd and 22C10 (E, F). The anterior end is up, midline marked by vertical lines. One to a few segments are shown. See text for the penetrance of the defects. **(A):** In control embryos, the MP2 divides to generate a dMP2 and a vMP2, located just below the MP1 pair (MP1 cells originate from a different lineage). MP1 pairs are very close on either side of the midline, d/vMP2 are posterior to MP1 and slightly larger and farther from the midline. The scale bar is 5 μm. (**B**): In *pros* mutant embryos, Odd-positive cells are slightly larger than a dMP2 (arrow). No extra cells are seen in MP2 or MP1 lineages in the mutant. Instead, missing Odd-positive cells are seen in the mutant (arrowhead). The scale bar is 5 μm. (**C, D**): In both control and *pros* mutant embryos, Odd-positive MP2 cells are formed around 4 hpf (C). By 14 hpf (D), in the control, MP2 has divided into dMP2 (Odd-positive) and vMP2 (Odd-negative) in each hemisegment; in the mutant, a single large cell instead of the d/vMP2 pairs is seen (arrows). Hemisegments with missing Odd-positive MP2 lineage (or MP1) are also seen (arrowhead). The penetrance of these defects is partial but can be severe (last panel in D). The scale bar in panel C is 10 μm, in panel D is 5 μm (D). (**E, F**): The Odd negative, 22C10-positive vMP2 projects its axon anteriorly (long arrows); the Odd and 22C10 positive dMP2 projects its axon posteriorly (E). In *pros* mutant embryos, hemisegments with only a dMP2 or a vMP2 are often observed. The Scale bar represents 5 μm.

MP2 is an S1 NB (Fig 3C) formed around 4 hpf in row 4, column 1 [16–18]. It divides around 6.5 hpf to generate two post-mitotic neurons, vMP2, which has an anterior axon projection and is located ventral and anterior to its sibling neuron dMP2, which has a posterior axon projection and is located dorsal and posterior to vMP2 (Fig 3A, 3D and 3E). The expectation is that there will be multiple stem cell-type of divisions of MP2 in *pros* mutant embryos and consequently, multiple progeny cells. The immunohistochemistry of embryos with Odd showed that an MP2 is properly formed in *pros* mutant embryos around 4–5 hpf (Fig 3C). However, in older mutant embryos, there were no extra MP2, dMP2 or vMP2 cells; instead, the MP2 appears not to divide (Fig 3B, 3D and 3F). It appears that the undivided MP2 adopts a dMP2, or a vMP2 fate (Fig 3F). About 50% (n = 220) of the hemisegments had this defect. Such direct differentiation would not be unusual since direct differentiation of GMCs such as GMC1-1a or GMC4-2a occurs in loss of function mutants for *cyclin A*, *regulator of cyclin A* or *string* [41,42]. About 16% (n = 220) of the hemisegments in *pros* mutant embryos had no Odd-positive MP2 or dMP2 present (Fig 3B and 3D, arrowhead). These are likely hemisegments where MP2 differentiates into a vMP2 (Odd-negative), or the identity of the cell is affected since Pros also plays a role in cell identity specification [22,23]. It is unlikely an instance of dedifferentiation; if that is the case, the cells would have been (re)expressing Odd, and there would be additional cells in the MP2 lineage. That MP2 may directly differentiate into dMP2 or vMP2 in the mutant is further indicated by the Odd and 22C10 staining (Fig 3F). We observed cells with a dMP2 axon projection and with Odd expression without an accompanying vMP2 (Fig 3F, middle panels). We also observed Odd-negative cells with a vMP2 projection without any Odd-positive MP2 or dMP2 accompanying it (Fig 3F, last panels). These cells with axon projections are contrary to the idea that MP2 de-differentiate in *pros* mutants and behave as stem cells.

### MP2 fails to divide in *pros* mutant embryos

**Fig 4:**
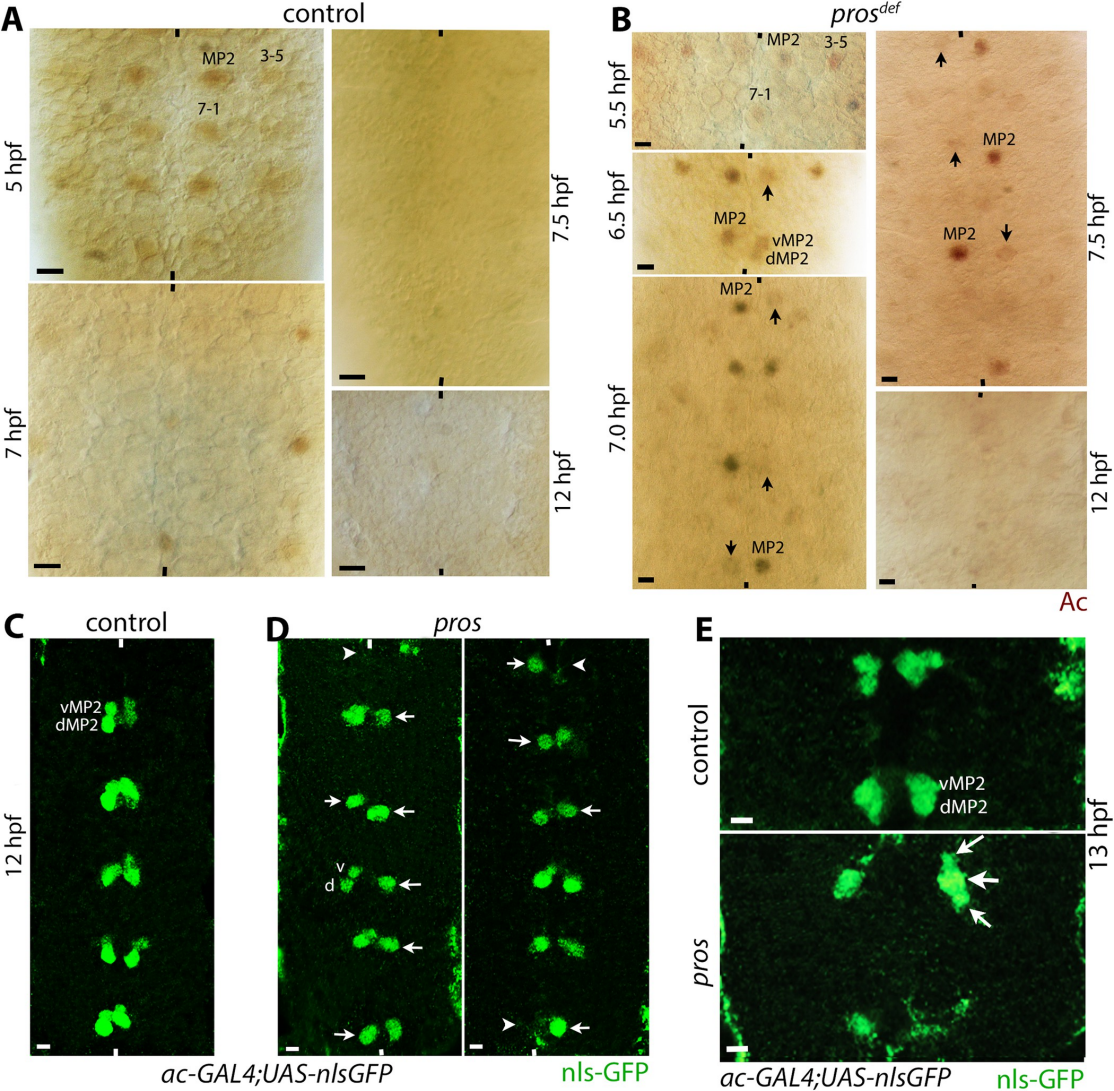
MP2 fails to divide in *pros* mutant embryos. Embryos are stained for Ac (panels A, B) and GFP (panels C, D). MP2 is formed around 4 hpf and divides around 6.5 hpf. Ac is present in MP2 and disappears from both vMP2 and dMP2 neurons during or soon after their formation. The anterior end is up, the midline is marked by vertical lines. The scale bar is 10 μm (A, B), and 5 μm (C-E). See text for the penetrance of the defects. **(A)**: The ventral nerve cord from wild-type control embryo showing Ac-positive MP2 in 5 hpf embryos. The Ac expression disappears from this lineage following MP2 division, and no Ac-positive cells in this lineage can be seen in >7 hpf embryonic nerve cord. **(B)**: The ventral nerve cord from *pros* mutant embryos. The Ac expression is present in MP2 in 5–6.5 hpf embryos, but also in an undivided MP2 in 7 or 7.5 hpf embryos. The undivided MP2 cells start to downregulate Ac around 7 hpf (arrow), and the Ac expression is undetectable in 12 hpf embryos. When an MP2 does divide in *pros* mutant embryos, the newly formed v/dMP2 cells retain Ac expression but at lower levels (second panel, lower-right hemisegment); the Ac expression disappears in these pairs by 7.5 hpf of age. **(C, D)**: Control (C) and *pros* mutant (D) embryos expressing nuclear-GFP in MP2 lineage. The UAS-nls-GFP transgene is induced with ac-GAL4 in wild-type control and in *pros* mutant backgrounds. Unlike Ac, nls-GFP is not degraded, therefore the MP2 cells can be traced into older stages of neurogenesis. In the control, GFP-positive v/dMP2 pairs per hemisegment are seen, whereas in the mutant, undivided MP2 cells (arrows) or missing MP2 (arrowhead) in hemisegments are seen. A minority of the hemisegments have the vMP2 and dMP2 formed. **(E):** A three-cell phenotype in *pros* mutant embryos is rarely observed. Control and *pros* mutant embryos expressing the nuclear-GFP in MP2 lineage. The UAS-nls-GFP is induced using ac-GAL4 in control and *pros* mutant backgrounds. In control, only dMP2 and vMP2 per hemisegment are seen, whereas in *pros*, a three-cell phenotype is seen (arrows), one larger, perhaps a dMP2 (arrow), and two smaller vMP2 cells (small arrows), generated from the same parent.

The possibility that MP2 fails to divide in *pros* mutant embryos was further examined. We used Achaete (Ac) as a second marker to stain *pros* mutant embryos. In wild-type, Ac is present in MP2 (Fig 4A) but disappears from vMP2 and dMP2 cells. In *pros* mutant embryos, we observed Ac-positive MP2 cells in 90% of the hemisegments (Fig 4B). However, unlike in wild-type control, Ac-positive MP2 cells were observed even at 7.5 hpf embryos (Fig 4B, bottom panel; 50% of the hemisegments, n = 30). MP2 should have been divided by then. Hemisegments with an MP2 where the expression of Ac was disappearing without cell division were also observed (40%, n = 30; Fig 4B, middle, bottom, and right-top panels). This indicates that MP2 is progressively differentiating directly into a neuron. Rarely, hemisegments where the MP2 has divided but still has residual Ac were also observed (Fig 4B, 6.5 hpf, middle panel). A previous report has suggested that in *pros* mutant embryos, cells dedifferentiate and start re-expressing parental genes [25]. Therefore, we examined *pros* embryos that were 12 hpf old to determine if these MP2 cells (or their progeny v/dMP2s) re-express Ac. No such re-expression of Ac was observed in these cells (Fig 4B).

To determine unambiguously if MP2 fails to divide in *pros* mutant embryos, a UAS-GFP with a nuclear localization signal tagged to GFP (UAS-nls-GFP) was introduced into the *pros* mutant background and was induced with the ac-GAL4 driver. The ac-GAL4 will induce nls-GFP in MP2. With the persistence of nls-GFP into older stages of development, we could track MP2 and its division pattern. As shown in Fig 4C, in control embryos, nls-GFP-positive vMP2 and dMP2 neurons were detected in every hemisegments (n = 20), whereas in *pros* mutant embryos, nearly 50% (n = 24) of the hemisegments had just one cell (Fig 4D, arrows). In about 15% of the hemisegments, no nls-GFP-positive cells could be seen (Fig 4D, arrowhead). In these lineage tracing experiments, interestingly, we also found 9% (n = 67) of the hemisegments in 13 hpf mutant embryos with 3 MP2-lineage originated cells (Fig 4E, arrows). These cells are unlikely dedifferentiated cells since we did not observe re-expression of Ac in *pros* mutant embryos in the MP2 lineage (Fig 4B), a crucial marker for dedifferentiation (see also below). Therefore, based on these results, we believe that in *pros* loss of function mutant embryos, MP2 fails to divide in a significant number of hemisegments. The presence of hemisegments with missing cells argue that cell identity may also be affected in *pros* mutant embryos, a finding consistent with the previous results [22,23]. In hemisegments where a three-cell phenotype was observed, the MP2 must have undergone an extra self-renewing division [17].

### No additional cells were observed in GMC4-2a and NB7-3 lineages in *pros* mutant embryos

**Fig 5:**
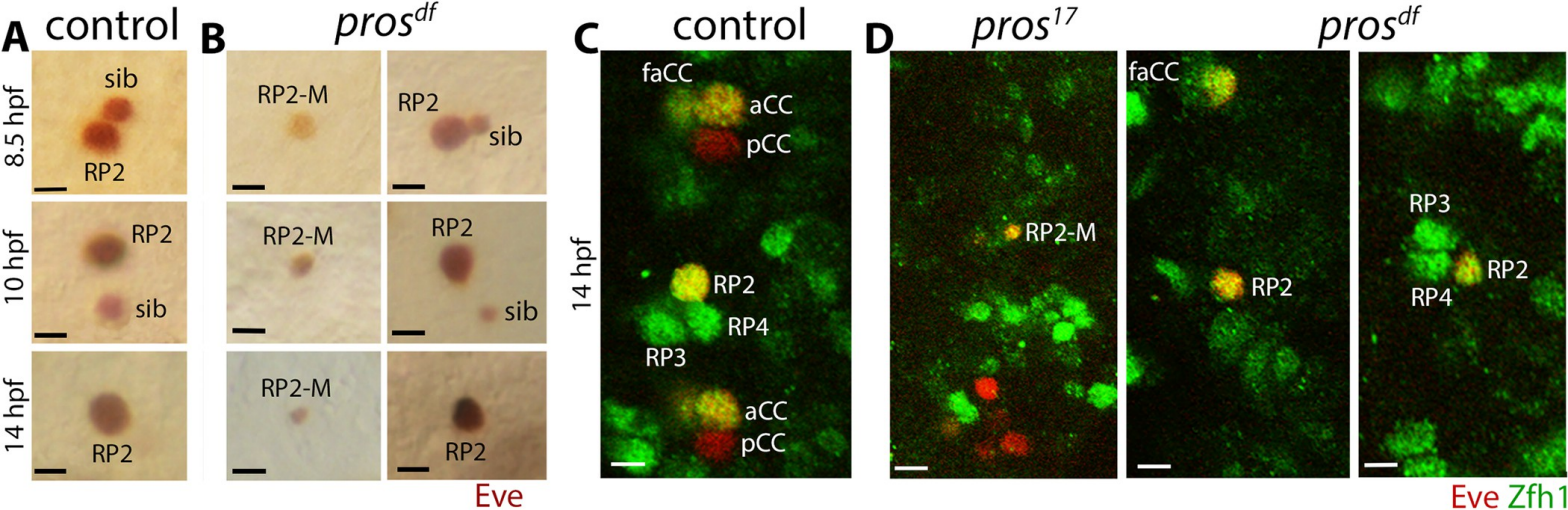
GMC4-2a fails to divide in *pros* mutant embryos. Control and *pros* mutant embryos are stained with anti-Eve (A), or double-stained with anti-Eve (Red) and anti-Zfh1 (Green)(B). The anterior end is up, the scale bar is 3 μm. See text for the penetrance of the defects. **(A):** In control, GMC4-2a has divided to generate a smaller sib and a larger RP2. The smaller sib gradually loses Eve expression, and in 14 hpf of age, only the RP2 retains Eve. **(B):** In *pros* mutant embryos, a GMC4-2a fails to divide and adopts an RP2 identity, a sib identity, or a mixed identity (RP2-M) with features of both RP2 and sib (middle panels). A hemisegment with normal development of the lineage is also observed in the mutant (right panels). **(C, D):** In control (C), Zfh1 positive RP2, RP3, and RP4 among the RP-cluster are seen. Zfh1 is also present in aCC and friend of aCC (faCC). RP2, aCC and faCC also have Eve expression. In *pros* mutant embryos (D), hemisegments with Zfh1-positive but smaller-sized sib-like cell (RP2-M), or a normal-sized RP2, but with lower levels of Zfh1, or a smaller but normal level of Zffh1 are seen. Missing RP3 or RP4 within the RP-cluster are also seen in the mutant.

We examined if the GMC4-2a lineage generates additional cells in *pros* mutant embryos. GMC4-2a is a well-studied lineage in the CNS [9,10,13,17,19,22,27,28]. In GMC4-2a, Pros is nuclear and mostly chromatin-bound (see Fig 1E). The GMC4-2a->RP2/sib (known as GMC-1->RP2/sib) is also a lineage we have been studying for many years. This GMC is formed from NB4-2 at around 6.5–6.45 hpf and divides around 7.5 hpf to give rise to a motor neuron called RP2 and its sibling cell, simply called sib (Figs 1E and 1F; 5A). In *pros* mutant embryos, only a few Eve-positive GMC4-2a could be seen (<5% of the hemisegments; n = 550), however, they rarely divided (<1%, n = 550) to generate an RP2 and a sib (Fig 5B, right panels), instead, they appear to adopt a mixed identity with the undivided cell being of a larger size and weak Eve-expression or small-sized sib-like cell with strong Eve expression (Fig 5B). These defects were observed in <4% of the hemisegments. Staining of mutant embryos with Zfh1, another antibody that identifies this lineage, also revealed similar findings (Fig 5D).

We next examined the NB7-3 lineage. This NB is an S5 NB, formed around 8–8.5 hpf in row 7, column 3. It divides 3 times to generate 3 GMCs: GMC7-3a, which divides to generate one serotonergic neuron and a GW cell, GMC7-3b, which generates another serotonergic neuron and a cell of unknown identity, and GMC7-3c, which generates a corazonergic neuron and another cell of unknown identity. NB7-3 and all its progeny (GMCs and neurons) express Eagle (Eg). We stained wild-type and *pros* mutant embryos with Eg antibody. While in wild-type NB7-3 is generating GMCs (S2 Fig, panel A2, right hemisegment), we did not observe cells that would account for such divisions in the mutant (S2 Fig, B1-B3). NB6-4 and NB2-4 also express Eg and are in close proximity to NB7-3; they also do not seem to generate extra cells in *pros* mutant embryos (S2 Fig, B1-B3). These defects were seen in >90% of the hemisegments (n = 72 hemisegments).

### Cyclin E is downregulated in early *pros* mutant embryos but elevated in older embryos

**Fig 6:**
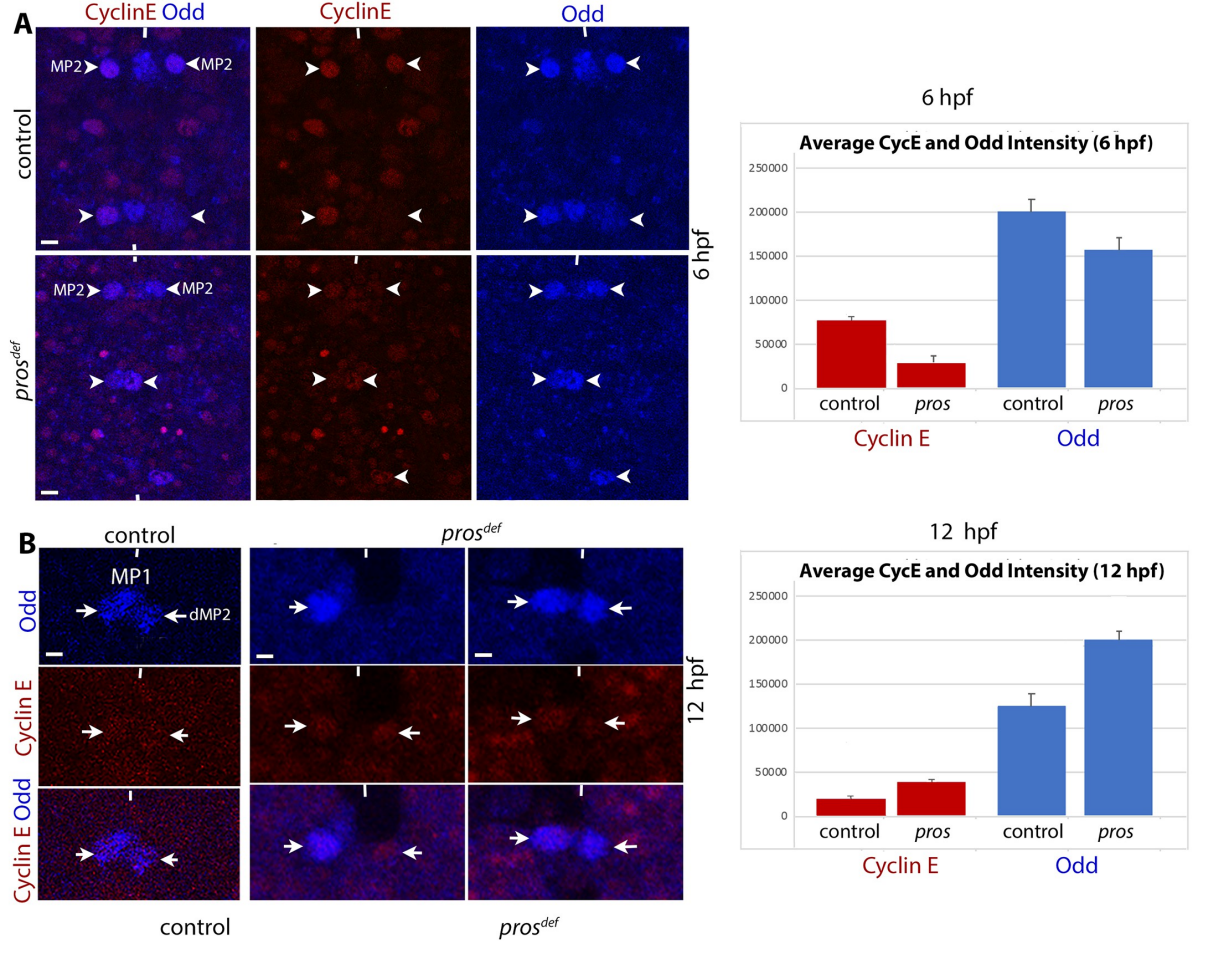
Mis-regulation of Cyclin E in MP2 lineage in opposite direction during development in *pros* mutant embryos. The anterior end is up, and the midline is marked by vertical lines. The scale bar is 10 μm. Cyclin E is red, Odd is blue. **(A):** Cyclin E is downregulated in MP2 in 6 hpf *pros* mutant embryos. Control and *pros* mutant embryos are double stained for Cyclin E and Odd (to identify MP2). MP2 on the lower right hemisegment is huge with diffused Odd and Cyclin E, which occurs just before its division [24]. Since the development between different hemisegments in any given embryo is slightly unsynchronized, one can see an MP2 before, during or a v/dMP2 pairs in the same embryo (there is a 15–25 min difference between hemisegments for MP2 lineage development; this window slightly varies between lineages). Quantification for Cyclin E and Odd expression is shown in the adjacent histogram. The difference in the levels of Cyclin E between control and *pros* mutants is statistically significant (P<0.05), whereas the levels of Odd between control and *pros* is not significant (P<0.1). The raw data used for the histogram is given in S3A Fig. We also analyzed the levels of Cyclin E and Odd in MP2 in a segment in control and the mutant using the plot profile function of ImageJ, which is another way to quantify the levels (S3B and S3C Fig). This analysis also showed a significant difference in the levels of Cyclin E in MP2 between the control and the mutant. **(B):** Cyclin E is upregulated in MP2 lineage in 12 hpf *pros* mutant embryos. Control and *pros* mutant embryos are double stained for Cyclin E and Odd. Quantification for Cyclin E and Odd expression is shown in the adjacent histogram. The difference in the levels of Cyclin E between control and *pros* mutants is statistically significant (P<0.001), the level of Odd is also statistically significant (P<0.05). See S4A Fig for the raw data used for the histogram. The plot profile analysis of the cells in these embryos also showed a significant difference in the levels of Cyclin E and Odd between the control and the mutant (S4B Fig).

Cyclin E plays a crucial role in the entry of cells to the cell cycle in the fly CNS [17,24,43–47]. Cyclin E is essential for the division of all neural precursor cells, stem cells included [17,24]. As shown in Fig 6, the failure of MP2 to divide in *pros* mutant embryos appears to be due to a reduction in the levels of Cyclin E. In the control embryos, the levels of Cyclin E were elevated in MP2 before its division (Fig 6A, 6 hpf, upper panels). In *pros* mutant embryos, as shown in Fig 6A (lower panels), the Cyclin E level was reduced in MP2 in about 60% of the hemisegments (n = 48). The difference in the levels of Cyclin E between control and *pros* mutants in 6 hpf was statistically significant (P<0.05), whereas the reduction in the levels of Odd in *pros* compared to control, was not statistically significant (P<0.1) (see also S3 Fig). This indicates that Pros augments Cyclin E levels in MP2 in early-stage embryos, and a reduction in the level in *pros* mutants may in part explain the lack of division of MP2 in *pros* embryos.

Next, we determined the levels of Cyclin E in the MP2 lineage in older stage embryos. As shown in Fig 6B, while in the control (left side panels) Cyclin E was hardly detectable, in *pros* mutant embryos, it was upregulated (Fig 6B, mid and right panels; see also S4 Fig). This result indicates that in later stages of development, Pros normally suppresses Cyclin E expression. The difference in the levels of Cyclin E between control and *pros* mutants in 12 hpf was statistically highly significant (P<0.001), and the levels of Odd between the control and *pros* mutants was statistically less significant (P<0.05). We think that the strong suppression of Cyclin E expression by Pros in later stages of neurogenesis likely contributes to the commitment of neurons to post-mitotic life history.

In lineage tracing experiments, we found that about 9% (n = 67) of the hemisegments in *pros* mutant embryos had 3 cells of MP2-lineage-origin (Fig 4E, arrows). These cells are not dedifferentiated cells (see Fig 4B). In these hemisegments, loss of function for *pros* must have resulted in the deregulation of Cyclin E in the opposite direction sooner to induce one more division. However, in majority of the hemisegments, the upregulation of Cyclin E in older *pros* mutant embryos was not sufficiently high enough for cells to re-enter the cell cycle.

**Fig 7:**
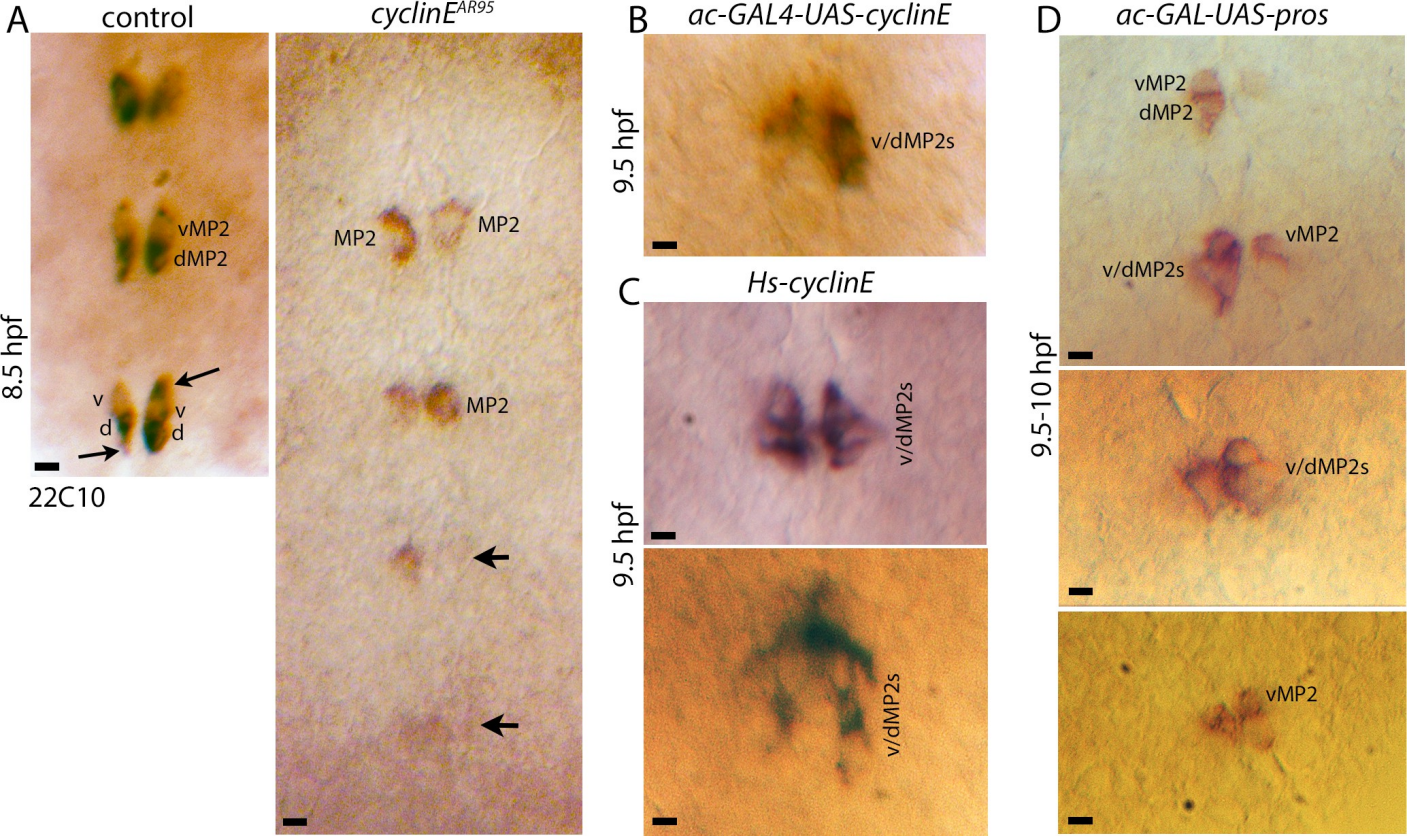
The effects of loss of function and gain of function for *cyclin E* and gain of function for *pros* in MP2 lineage. Embryos are stained for 22C10. The anterior end is up. The scale bar is 5 μm. See text for the penetrance of the defects. **(A):** Ventral nerve cord segments from control and *cyclin E* loss of function mutant embryos. In control, hemisegments with vMP2 and dMP2 neurons are seen with axons starting to sprout from the cells (arrow). In the mutant, hemisegments with a single undivided MP2 expressing the neuronal (d/vMP2)-specific 22C10 are seen; MP2 in some hemisegments have a low or no expression of 22C10 (arrow). **(B, C):** Ventral nerve cord segments from *cyclin E* gain of function embryos. In panel B, a *UAS-cyclin E* transgene is induced in MP2 with ac-GAL4; in panel C, heatshock70 promoter-linked *cyclin E* is induced with a brief heat shock. The hemisegments have additional v/dMP2 cells in these embryos. **(D):** Segments from the ventral nerve cord of *pros* gain of function embryos where a *UAS-pros* transgene is induced in MP2 with ac-GAL4. Multiple cells are seen in the MP2 lineage in these hemisegments.

It is also that in older stage embryos, cells have already differentiated with axon projections, therefore, despite an elevated Cyclin E no such divisions are possible. This contention is also supported by the previous findings that overexpression of Cyclin E induced neuronal precursors to undergo additional rounds of cell division [17, 24]; however, over, or ectopic expression in post-mitotic neurons did not induce their cell division [24]. Nonetheless, to directly test the role of Cyclin E in MP2 division, we examined MP2 lineage in embryos mutant for *cyclin E*. As shown in Fig 7A with Mab22C10 (MAPIB) staining, while in the wild-type control, vMP2 and dMP2 neurons were present in 98% of the hemisegments (N = 48), in *cyclin E* mutants, we observed hemisegments with missing 22C10-positive cells (Fig 7A, arrows), weakly stained cells, and hemisegments with a larger-sized cell (compared to its progeny) that are 22C10-positive (Fig 7A). About 29% (n = 48) of the hemisegments showed these defects. MP2 cells do not express 22C10, but only its progeny neurons do. These results suggest that MP2 in *cyclin E* mutants may also adopt one of its sibling fates.

We next examined the effects of overexpression of Cyclin E in MP2 using a *UAS-cyclin E* transgene-induced with ac-GAL4. As shown in Fig 7B, extra divisions in the MP2 lineage with additional v/dMP2 cells were observed. This phenotype was not very penetrant since only about 9% (n = 96) of the hemisegments had this phenotype. It is very likely that the control of Cyclin E levels is very robust, and despite its over-expression, the protein gets degraded. Besides, the expression of ac-GAL4 is expected to be switched off as the MP2 divides. Therefore, we over-expressed Cyclin E using a *cyclin E* transgene under the control of the *Heat shock 70* gene promoter [17]. While the expression from the *Hs-cyclin E* transgene is temporally restricted, the level should be much higher than when the *UAS-cyclin E* was induced with the ac-GAL4. The *Hs-cyclin E* transgene was induced in these embryos by incubating the transgenic embryos at higher temperatures (see Materials and Methods), and the MP2 lineage was examined by staining them with the 22C10 antibody. As shown in Fig 7C, 3–4 cells per hemisegments were observed in these embryos. This phenotype was observed in as many as 60% (n = 96) of the hemisegments in the affected embryos.

Finally, we also examined if the development of the MP2 lineage is altered by the over-expression of Pros in MP2. We induced *UAS-pros* with ac-GAL4 and examined the MP2 lineage with 22C10 antibody. As shown in Fig 7C, over-expression of Pros resulted in the generation of multiple cells in the MP2 lineage. This defect was seen in about 16% (n = 28) of the hemisegments in the affected embryos. The lower penetrance of the defects likely reflects a tight organismal control of the levels or the pathways. Indeed, over/ectopic expression of most genes does not necessarily produce a phenotype, or a phenotype that is the same or opposite to that of loss of function, therefore, the low penetrance is not entirely unexpected. The fact that it does produce an opposite phenotype of loss of function for *pros* is consistent with the conclusion that a correct dosage or level of Pros confers the single division potential to MP2.

## Discussion

What makes precursor cells divide a certain number of times and how the progeny cells become post-mitotic has remained enigmatic. These questions are among some of the most fundamental questions in neurobiology. The work described here provides a clue and indicates that Pros and Cyclin E may be some of the key players in these processes. Our results indicate that the cytoplasmic, non-phosphorylated Pros becomes phosphorylated and nuclear and binds to chromatin in cells destined to divide once. It must directly or indirectly regulate gene expression in the nucleus. One such gene regulated by Pros appears to be Cyclin E. Ample data indicates that Cyclin E is essential but also sufficient to drive entry of precursor cells to the cell cycle [17,24,43–47], although within a temporal window of developmental time [24,17]. The data presented here show that Pros regulates Cyclin E levels in opposing directions between precursor cells and their progeny. This is an elegant, yet simple mechanism by which Pros through Cyclin E confers the one-division potential to MP2, GMC4-2a, or GMCs from NB7-3, and then helps commit their progeny to a post-mitotic state. How many lineages in the CNS also utilize this mechanism remains unknown.

**Fig 8:**
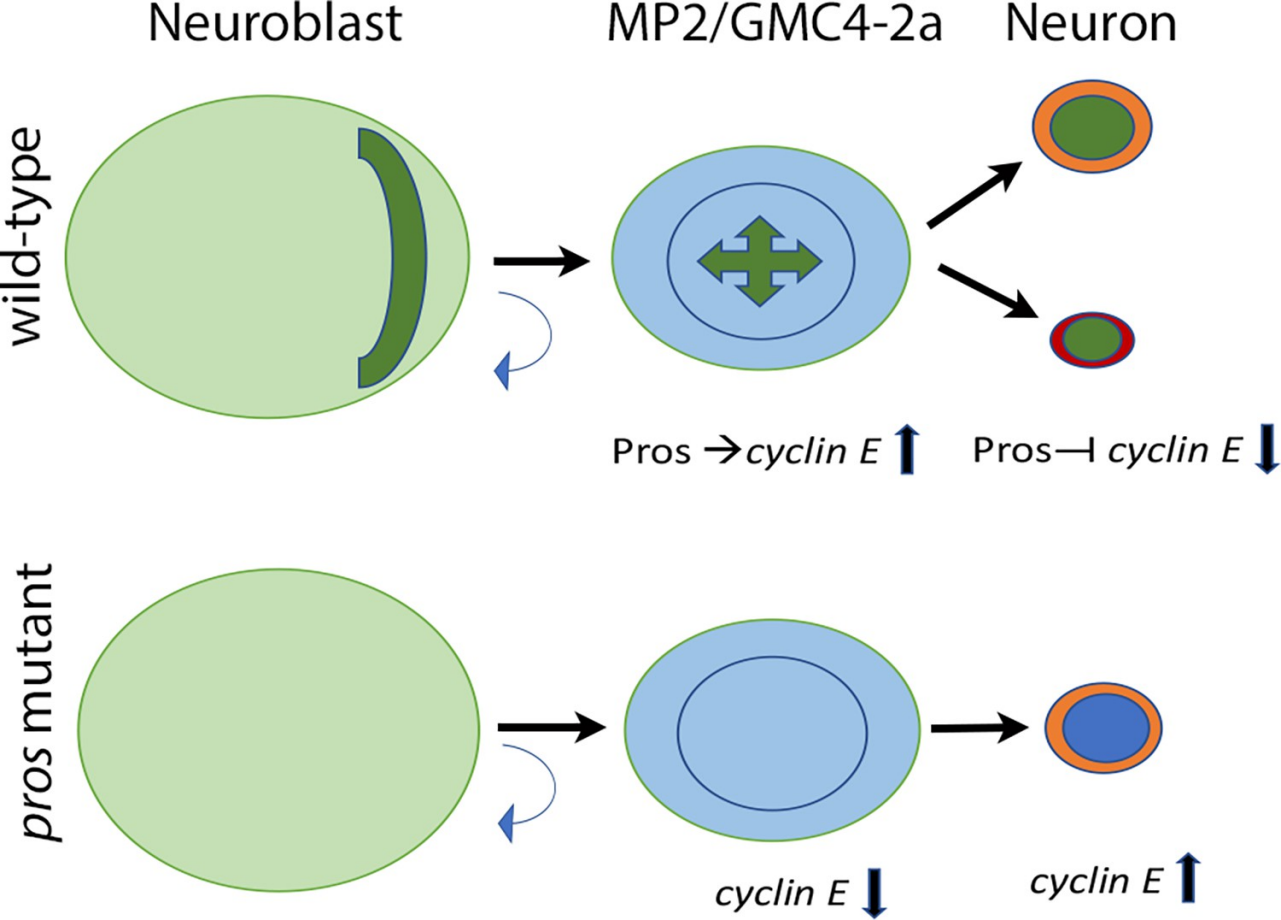
Pros mediates the division potential of precursors and post-mitotic status of neurons through opposing regulation of Cyclin E. In wild-type, Pros is cytoplasmic in NBs, but bound to chromatin in MP2 or GMC. In MP2 and GMC4-2a (or NB7-3), Pros augments the upregulation of expression of Cyclin E to promote a single division. It augments the downregulation of Cyclin E in progeny neurons, committing them to their post-mitotic fate. Thus, Pros restricts the division potential of precursors (a single) and neurons (none) through Cyclin E up or down regulation.

The situation is not an ON/OFF scenario. A clear ON/OFF scenario will also be evolutionarily prohibitive as it would negatively affect the neuronal number, plasticity, and diversity. Instead, Pros appears to augment the upregulation of Cyclin E level in the precursor enough to commit that cell to divide once (Fig 8). Once it divides to generate two daughters, Pros augments the downregulation of levels of Cyclin E such that progeny cells do not enter the cell cycle, but instead become post-mitotic (Fig 8). The evidence to support this model comes from the fact that in *pros* loss of function mutants, cells such as MP2 and GMC4-2a fail to divide (Figs 3–5; S2). The levels of Cyclin E were also downregulated in MP2 in *pros* mutant embryos (Figs 6A and S3), indicating a positive role for Pros via augmenting Cyclin E expression in MP2 division. However, in *pros* loss of function mutants at a later developmental stage, the level of Cyclin E was upregulated (Figs 6B and S4), indicating a repression role for Pros in older stage embryos. Thus, a repression of Cyclin E by Pros could lead to the post-mitotic status of progeny neurons. A switch from an activator to a repressor can be achieved by partnering with different transcription regulators before and after cell division. These results are further supported by the finding that MP2 fails to divide in loss of function for *cyclin E*, and gain of function for *cyclin E*, or gain of function for *pros* leads to extra divisions (Fig 7). The penetrance of these defects is not very high, but we think that this is to be expected since players such as Cyclin E will be tightly regulated during development. There is also the issue of maternal deposition when loss of function mutations is in question.

The opposing role of Pros in *cyclin E* regulation, depending upon the developmental stage, is meant to switch from facilitating a single division of precursors to facilitating a post-mitotic commitment of progeny cells. De-repression of Cyclin E alone in progeny cells in late-stage embryos either in the *pros* mutant (this study) or by over-expression of Cyclin E in post-mitotic progeny cells [17,24, this study] does not appear to be sufficient to make them re-enter the cell cycle. It may be that the Cyclin E level was not high enough in those stages of development, or the process that makes cells post-mitotic involves additional players. Thus, elevating Cyclin E alone at the “Cyclin E-insensitive” stage may not be enough to make them re-enter cell cycle. Additionally, differentiation genes also begin to express in progeny cells, and then there is the physical process of differentiation that gets underway with neurites sprouting and axon forming. These structural changes in post-mitotic neurons could also prevent them, in addition to new gene expression programs, from re-entering the cell cycle despite elevated levels of Cyclin E. Furthermore, in 12 hpf or older *pros* mutant embryos, there is a general up-regulation of Cyclin E, not only in the MP2 lineage, but also in other cells in the nerve cord. We do not know the consequence of this upregulation such as if those lineages produce extra cells. We are in the process of examining this question. We also do not know if Pros plays a similar role in type II NBs in the embryonic nervous system or during neurogenesis of the adult brain.

These results also argue that there may not be a dedifferentiation of cells in *pros* mutants as previously thought [25], at least not in every lineage. Pros, with its chromatin localization in cells committed to a differentiation pathway, appears to control many genes. Cyclin E alone, at least in earlier stages of development, is sufficient to make cells divide or not divide depending on the levels, and Pros fits in this Cyclin-E-mediated model by its ability to regulate *cyclin E* expression. In how many lineages Pros confers the single-division potential to precursor cells is not clear. We also do not know if the asymmetrically localized cytoplasmic Pros in NBs has any role in cell division or if it simply is a mechanism to segregate Pros to GMCs. In any event, Pros is unlikely to regulate Cyclin E in NBs (other than MP2/NBs that have single division potential).

Finally, these results are consistent with our previous finding that in embryos mutant for a gene called *midline*, MP2 undergoes multiple self-renewing asymmetric divisions. Pros was cytoplasmic in MP2 in *midline* mutants [24], which further indicates that the single division potential of MP2 correlates with a nuclear/chromatin-bound Pros. A recent paper indicated that Pros remodels H3K9me3+ pericentromeric heterochromatin by recruiting Heterochromatin Protein 1 during neuronal differentiation [39]. This conclusion is consistent with our supposition that Pros augments post-mitotic commitment and neuronal differentiation of progeny cells, and regulation of Cyclin E and modulating heterochromatin are essential to these developmental events. Neuronal differentiation is a complex and evolutionarily crucial process for survival; therefore, it is not surprising that various mechanisms will augment the process as a shared phenomenon. A partial redundancy for a gene or a pathway is a common theme during neurogenesis or development [48].

## Materials and methods

### Fly stocks, genetics

We used the following fly stocks in this study: *UAS-GFP*.*nls* (2^nd^ chromosome, BDSC 4775 [49], *ac-GAL4* (3^rd^ chromosome, BL# 8715); for the analysis of *pros* loss of function, we used a *pros-*deficiency [Df(3R)Exel7308; breakpoints: 86D9-86E5; BL#7959] and *pros*^*17*^, which is a loss of function allele (BL#5458), both were rebalanced with a GFP-marked 3^rd^ chromosome balancer (*kruppel*-GFP; BL#5195) since *pros* is located on the 3^rd^ chromosome. The *pros* gene is located at 86E2-86E4. The other lines used were: *UAS-cyclin E* (BL# 4781) and *Hs-cyclin E* (BL# 59060). For control, we used Oregon-R and or Canton-S; when a GAL4 driver was involved, we also used the driver line as another control. All lines were balanced with a GFP-marked balancer chromosome(s) to help identify homozygous mutant embryos. The following stock was made by standard genetics: *UAS-GFP-nls/Cyo; pros17*, *ac-GAL4/TM3 Sb*. These flies were mated with flies of genotype *pros*^*17*^*/ TM3 Kr-GAL4*, *UAS-GFP*, and embryos were collected and stained for Pros (to help identify the homozygous mutant embryos) and GFP. For the analysis of the MP2 lineage in *cyclin E* mutants, we used *cyclin E*^*AR95*^, a loss of function allele. Unless otherwise stated, experiments were done at room-temperature.

### Immunohistochemistry

The embryo collection, fixation, and immunostaining were performed according to the standard procedures. Briefly, for immunolabeling, embryos were washed thoroughly with running water, dechorionated with 50% bleach, rinsed with running water, and then with phosphate-buffered saline containing Triton X-100 (Sigma) (0.1%), and fixed with n-heptane (Fisher Scientific) and 37% formaldehyde (Fisher Scientific) mixed in a 1:1 ratio for 2 min (for immunofluorescence labeling) or 6 min (for immunohistochemical labeling). Vitelline membranes were removed by a rapid (~20 seconds) wash with methanol (Fisher Scientific). Embryos were processed immediately in most cases. The following antibodies were used: anti- Pros (mouse, 1:5, DHSB), anti-Fas II (mouse, 1: 10; DHSB), 22C10 (mouse, 1:1; DHSB), BP101 (1:10, mouse, DHSB), anti-Achaete (mouse, 1:1; DHSB), anti-GFP (chicken, 1:300, Novus Bio), anti-Odd (guinea pig, 1:100, John Reinitz), anti-Cyclin E (1:4; source: Helena Richardson), Eve (1:2000; Manfred Frasch), and anti-Zfh1 (1: 4; Eric Lai). For color visualization, either AP-conjugated or HRP-conjugated secondary antibodies were used. For light microscopy, secondary antibodies conjugated to alkaline phosphatase (rabbit, 1:200, Pierce, 31341) or horseradish peroxidase (HRP; rabbit, 1:200, Pierce, 31460) were used. Alkaline phosphatase was detected using 5-bromo-4- chloro-3-indolyl-phosphate and nitro blue tetrazolium (Promega, S3771). HRP was detected with diaminobenzidine (Sigma, D4418). For confocal visualization, the following secondary antibodies were used: Goat anti-Mouse Alexa Fluor 488 (Invitrogen, 1:300), Goat anti-chicken Alexa Fluor 633 (Invitrogen, 1:300), or Alexa Fluor 647 (rabbit or mouse, 1:300, Invitrogen, A-21245 or A-21236).

### Over/Ectopic expression of *cyclin E* and *pros*

For the overexpression of *cyclin E* in MP2 lineage, a *UAS-cyclin E* and a *HS-cyclin E* transgenic lines [17,24] were used. For the over-expression of *pros*, a *UAS-pros* transgenic line was used. In *UAS-Cyclin E* and *UAS-pros* cases, *ac-GAL4* was used to induce the genes in the MP2 lineage. The *Hs-cyclin E* experiments were done as follows: embryos from *Hs-cyclin E* were collected over a four-hours period and then aged for an additional four hours on apple juice plates at 25°C (these embryos are 4–8 hpf). Heat shock was induced by incubating these embryos at 37°C for 30 minutes. Embryos were then moved back to 25°C and allowed to recover for two hours. Our target embryos were around 6–7 hpf for the induction (when MP2 is dividing); after induction and aging, the target embryos for analysis were around 9–10 hpf. Heat-shocked embryos were dechorionated in bleach and fixed in heptane:formaldehyde at 1:1 ratio for six minutes. Immunohistochemistry with 22C10 was performed as described above with color development using HRP or AP-reactions.

### Western analysis

Western blot analysis was done as follows. Control and mutant embryos were collected at room temperature (25°C), dechorionated, and washed with running water. The GFP-negative mutant embryos were selected using an ultraviolet (UV) light–equipped Zeiss microscope since the mutant line was balanced with a GFP-labeled balancer chromosome. The embryos were lysed in chilled 40 ul of extraction buffer [150 mM NaCl, 0.5% sodium deoxycholate, 0.1% SDS, 1% NP-40, 50 mM Tris-HCl, pH 8.0, supplemented with PMSF (Final Concentration: 1 mM, Sigma Aldrich 78830) and PIC (Final Concentration: 1x, Sigma Aldrich P2714)]. The lysis was done by sonication for 1 min on ice in a 1.5-ml Eppendorf vial using a handheld sonicator (Thermo Fisher Scientific) equipped with a disposable pestle (Thermo Fisher Scientific). The lysates were centrifuged for 5 min at 13,000 rpm in a microfuge (Beckman). The supernatant was then transferred to a clean 0.6mL tube on ice and mixed thoroughly with an equal volume of 2x LDS Sample Buffer and Reducing Agent (Invitrogen). The samples were then heated to 95°C for 5 minutes and stored at -20°C until use. About 40 embryos equivalent amounts were loaded per lane on a 3–8% Tris-Acetate Gel (Invitrogen EA0375BOX), and proteins were transferred to a 0.2μM nitrocellulose membrane (Bio-Rad) using a Trans-Blot Turbo Transfer System (Bio-Rad). Following the transfer, membranes were incubated in 25 mL Ponceau S Stain (Sigma Aldrich P7170) for 5 minutes. Following incubation membranes were extensively washed with water, and protein bands present were imaged and used as a loading control. Membranes were then blocked in 25 mL 1x TBST (Tris Buffered Saline 0.1% Tween-20) + 5% powdered milk for one hour at room temperature. Membranes were then extensively washed to remove the excess block and incubated in 5 mL of primary antibody solution which consisted of the primary antibody diluted in 1x TBST + 1% powdered milk. Incubation in the primary solution was carried out overnight at 4°C on a BenchRocker (Benchmark Scientific). The primary antibody solutions were removed, and the membranes were washed for 3 x 5 minutes in 1xTBST. Following the third wash, membranes were incubated in respective secondary antibody solutions. All secondary antibodies were diluted in 1x TBST + 1% powdered milk to a final volume of 5 mL. The membranes were incubated in the secondary solutions for 2 hours at room temperature on a BenchBlotter 2D Rocker (Benchmark Scientific). Following this, they were washed for 3 x 5 minutes in 1xTBST to remove excess antibodies. Membranes were then incubated in Immobilon Western Chemiluminescent HRP Substrate (MilliporeSigma) according to the manufacturer’s directions and imaged using an Amersham Imager 600 (GE Healthcare Life Sciences). Primary antibodies used were against Pros (MR1a, mouse, 1:3, DSHB) and Pros (P3D4, rat, 1:2, Abcam), and the secondary antibodies were HRP-conjugated Goat anti-Mouse and Goat anti-Rat (1:7500, Jackson Laboratories).

### Dephosphorylation assay

Timed collection of embryos was dechorionated in a 1:1 bleach solution (LabChem, Inc. LC246302) for two minutes. Embryos were then extensively rinsed with water for at least 2min. A 10x Lysis Buffer (100 mM Tris pH 8, 100 mM NaCl, 10% NP-40) was prepared beforehand, diluted to 1x with sterile water, and chilled on ice. Embryos were counted under a stereomicroscope and 225 embryos were transferred to a 1.5 mL microcentrifuge tube containing 80 μL 1x chilled lysis buffer supplemented with 100x EDTA-free PIC (Final Concentration: 1x, Prometheus Cat #: 18–420). Embryos were then homogenized for one minute on ice using a hand-held homogenizer (Bel-Art) and plastic pestle (Fisher Scientific). The homogenate was then centrifuged at 13,000xg for 5 minutes at 4°C (Beckman). The lysate was then split into two 40 μL aliquots, one control and one test lysate, distributed to 0.6 mL tubes on ice. To the control lysate, a 10% SDS solution (Final Concentration: 1% Bio-Rad 1610301) was added along with 44 μL 2x LDS Sample Buffer + Reducing Agent (Invitrogen NP0007 & NP0004). The sample was then heated to 95°C for 5 minutes and then stored at -20°C until further use. To the test lysate, 5 μL of both 10x Lambda Protein Phosphatase Buffer and 10x MnCl_2_ were added followed by 1 μL Lambda Protein Phosphatase (Sigma Aldrich P9614). The sample was then incubated in a water bath at 30°C for 30 minutes. After this first incubation, 1 μL of Alkaline Phosphatase (Sigma Aldrich P0114) was added to the sample and incubated in a water bath at 37°C for 15 minutes. Following this a 10% SDS solution (final concentration: 1% Bio-Rad 1610301) was added along with 68.2 μL 2x LDS Sample Buffer + Reducing Agent (Invitrogen NP0007 & NP0004). The sample was then heated to 95°C for 5 minutes and stored at -20°C before performing the Western analysis along with the control.

### ImageJ analysis

We analyzed the expression of Cyclin E and Odd (Fig 6A and 6B) using the ImageJ software in two different ways. First, to measure the intensity of Cyclin E in MP2/dMP2 cells in 6 hpf and 12 hpf embryos, we used the Fiji/Image J software. Cells were marked with circles based on Odd staining for pixel analysis for both Cyclin E and Odd (Fig 6, histograms; S3A and S4A Figs). Cyclin E fluorescence intensity was measured for the same size. The background intensity of a neighboring circular area also of the same size was subtracted from the integrated density value for each cell analyzed. This value is the corrected total cell fluorescence. Second, we also used the plot profile function to determine the level-distribution profile of Odd and Cyclin E (S3B, S3C and S4B Figs). The following steps were adopted: images were saved as 300-pixels/Inch resolution in Adobe Photoshop, and then converted into JPEG files. The JPEG images were opened with ImageJ with the following measurement setup: Area, mean gray value, and integrated density under “Measurements”. Using the rectangle function, the area for plot profile analysis was defined, and under the function “Analyze”, the Plot profile was done.

### Statistical analysis

Unpaired student t-test was used to determine statistical significance of differences.

## Supporting information

S1 Fig The X-chromosome localization of H4K16Ac in the Drosophila male nucleus. Wild-type embryonic GMCs (A), 3^rd^ instar larval fat body (B), and 3^rd^ instar male (C) and female (D) larval salivary glands are stained for H4K16Ac. H4K16Ac is specifically localized to the X-chromosome in males [see also ref. 42]. We could not do a combination of Pros and H4K16Ac antibody staining as they prevented proper staining of each of the antigens. The scale bar is 5 μm (A, B) and 30 μm (C, D).
(TIF)

S2 Fig Fewer cells in the NB7-3 lineage in *pros* mutant embryos. Control and *pros* mutant embryos are stained with an anti-Eagle (Eg) antibody. The anterior end is up. (**A1-A3)**: Control embryonic ventral nerve cord with three different focal planes of the same embryo showing one segment. In the control embryo, NB7-3 divides at least once by 9.5 hpf of development (shown in panel A3). (**B1-B3):** *pros* mutant embryonic ventral nerve cord with three different focal planes of the same embryo showing three segments. There are fewer, and not additional, cells in *pros* mutant embryos in this lineage. No developmental delays are seen in *pros* mutant embryos which might account for the fewer cells in the lineage. The scale bar is 10 μm.
(TIF)

S3 Fig Data for the quantification of levels of Cyclin E and Odd in 6 hpf control and *pros* mutant embryos. Refer to Fig 6A. **(A):** Pixel intensity analysis within the circle for Cyclin E (shown) and Odd in MP2 cells in control and *pros* mutant embryos. See the methods section for details. The raw data and the average intensity with the standard error are shown in the tables, see materials and methods and the legend for Fig 6 for statistics. **(B, C):** Quantification of levels of Cyclin E in MP2 in 6 hpf control and *pros* mutant embryos using the plot-profile analysis of ImageJ. The plot profile is obtained within the boxed area and shown on the right. The arrowhead on the plot profile marks the expression profile for MP2s. The reduced expression of Odd in the control in the middle segment is likely a staining artifact.
(TIF)

S4 Fig Data for the quantification of levels of Cyclin E and Odd in 12 hpf control and *pros* mutant embryos. Refer to Fig 6B. **(A):** Pixel intensity analysis of Cyclin E and Odd in MP2 cells in control and *pros* mutant embryos. The raw data and the average intensity with the standard error are shown in the tables, see materials and methods and the legend for Fig 6 for statistics. **(B):** Quantification of levels of Cyclin E in MP2 lineage in 12 hpf control and *pros* mutant embryos using the plot-profile analysis of ImageJ. The plot profile is obtained within the boxed area and shown on the right. The arrowhead on the plot profile marks the expression profile for dMP2 in control and the undivided MP2-lineage cell in the mutant. The missing Odd-positive cell in the mutant (right panel) could be due to the direct differentiation of MP2 to vMP2 (an Odd-negative cell), or a staining artifact.
(TIF)
