# Supplementary figures and images for: Nuclear Prospero allows one-division potential to neural precursors and post-mitotic status to neurons via opposite regulation of Cyclin E
Source: PLoS Genet. 2022 Aug 8;18(8):e1010339. doi: 10.1371/journal.pgen.1010339 (PMC9359583; doi:10.1371/journal.pgen.1010339)

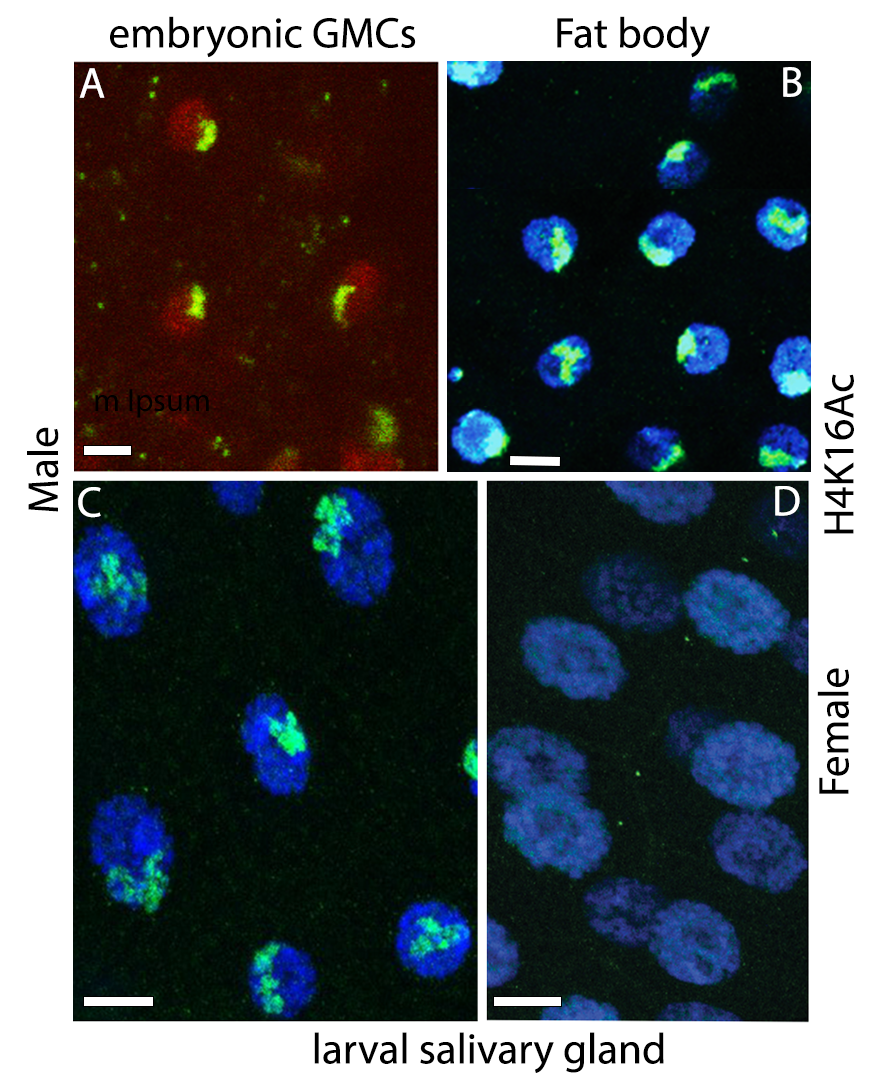

Supplement: S1 Fig — Wild-type embryonic GMCs (A), 3rd instar larval fat body (B), and 3rd instar male (C) and female (D) larval salivary glands are stained for H4K16Ac. H4K16Ac is specifically localized to the X-chromosome in males [see also ref. 42]. We could not do a combination of Pros and H4K16Ac antibody staining as they prevented proper staining of each of the antigens. The scale bar is 5 μm (A, B) and 30 μm (C, D). (TIF) [file pgen.1010339.s001.tif]

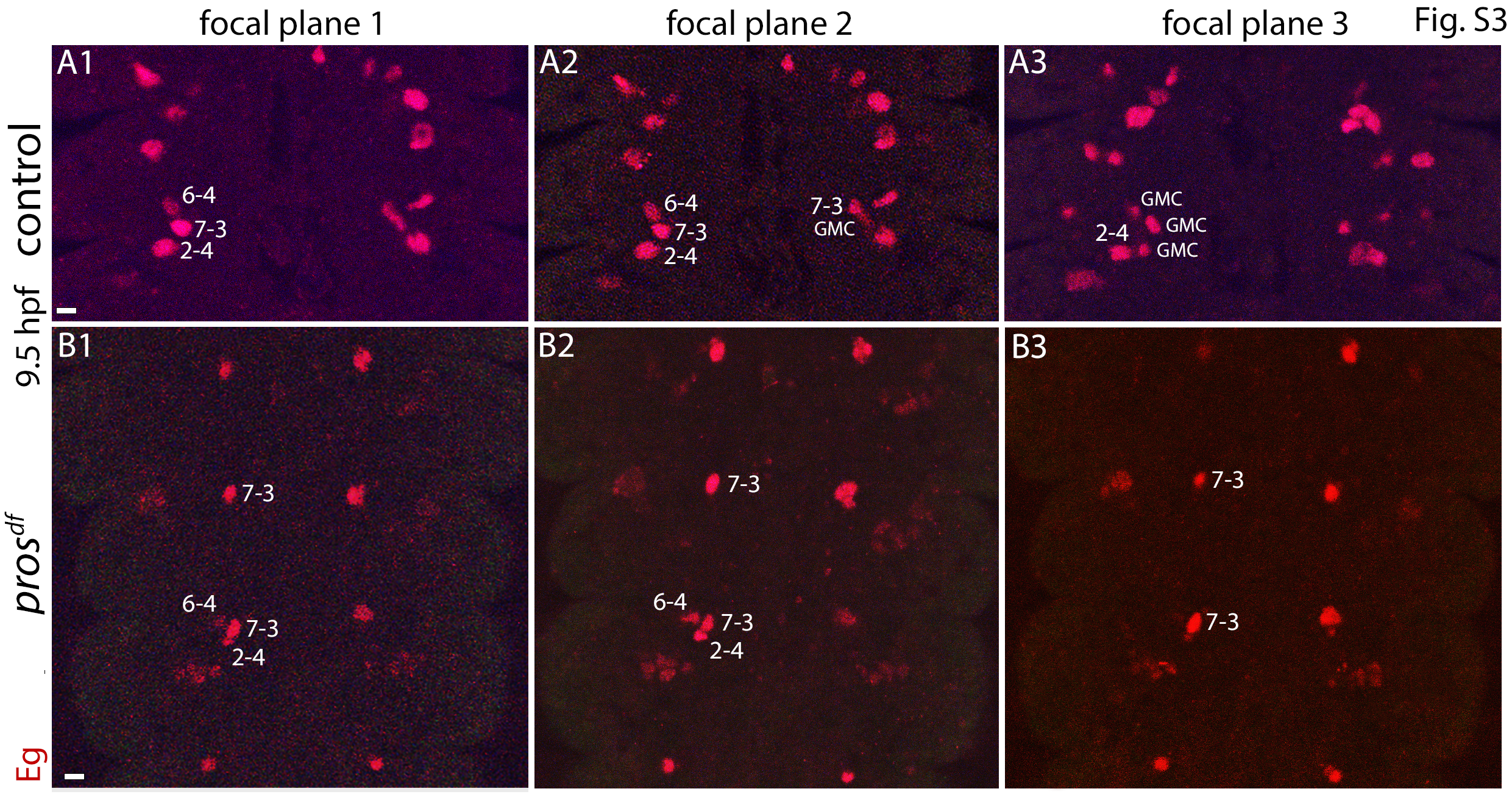

Supplement: S2 Fig — Control and pros mutant embryos are stained with an anti-Eagle (Eg) antibody. The anterior end is up. (A1-A3): Control embryonic ventral nerve cord with three different focal planes of the same embryo showing one segment. In the control embryo, NB7-3 divides at least once by 9.5 hpf of development (shown in panel A3). (B1-B3): pros mutant embryonic ventral nerve cord with three different focal planes of the same embryo showing three segments. There are fewer, and not additional, cells in pros mutant embryos in this lineage. No developmental delays are seen in pros mutant embryos which might account for the fewer cells in the lineage. The scale bar is 10 μm. (TIF) [file pgen.1010339.s002.tif]

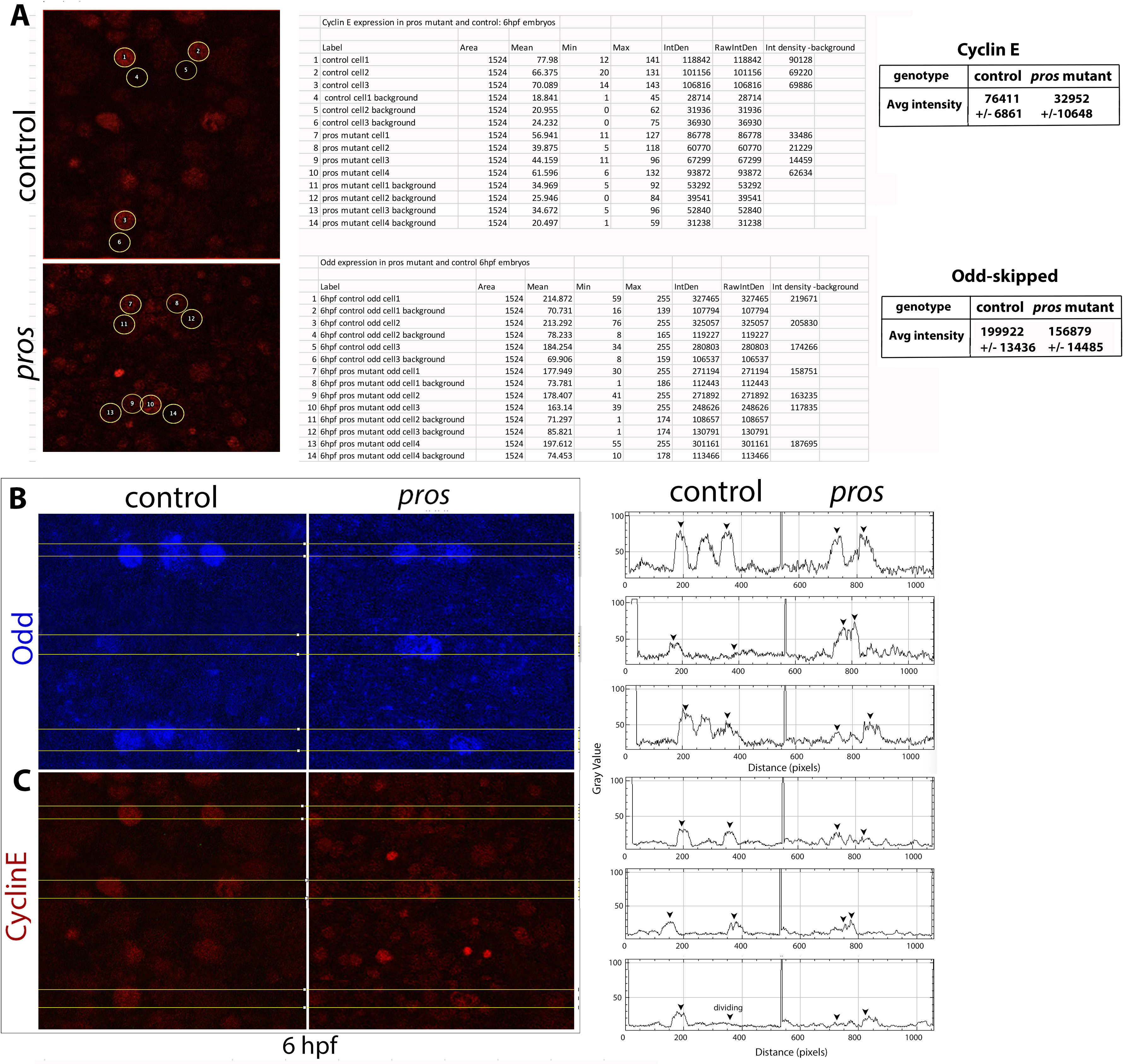

Supplement: S3 Fig — Refer to Fig 6A. (A): Pixel intensity analysis within the circle for Cyclin E (shown) and Odd in MP2 cells in control and pros mutant embryos. See the methods section for details. The raw data and the average intensity with the standard error are shown in the tables, see materials and methods and the legend for Fig 6 for statistics. (B, C): Quantification of levels of Cyclin E in MP2 in 6 hpf control and pros mutant embryos using the plot-profile analysis of ImageJ. The plot profile is obtained within the boxed area and shown on the right. The arrowhead on the plot profile marks the expression profile for MP2s. The reduced expression of Odd in the control in the middle segment is likely a staining artifact. (TIF) [file pgen.1010339.s003.tif]

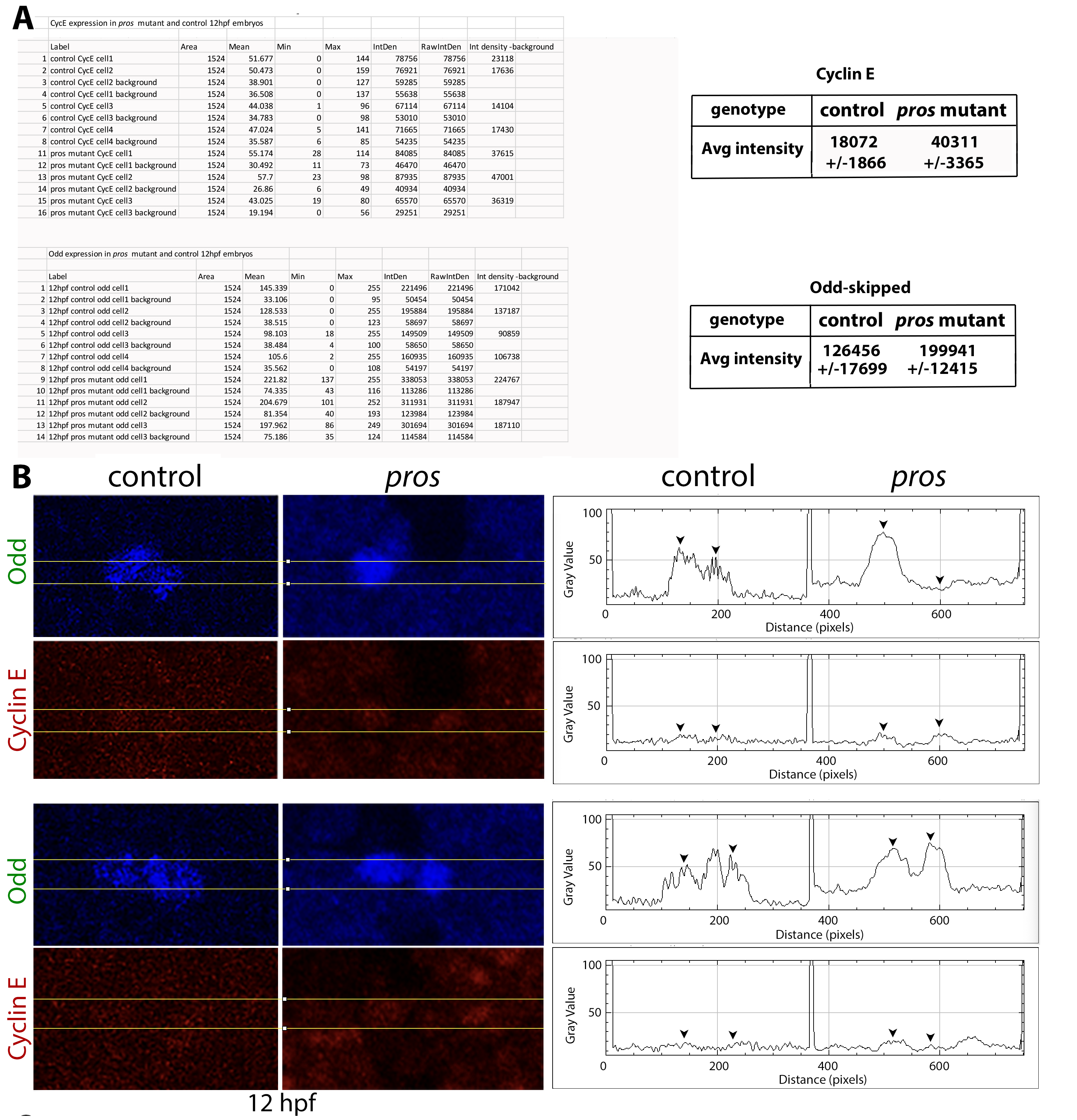

Supplement: S4 Fig — Refer to Fig 6B. (A): Pixel intensity analysis of Cyclin E and Odd in MP2 cells in control and pros mutant embryos. The raw data and the average intensity with the standard error are shown in the tables, see materials and methods and the legend for Fig 6 for statistics. (B): Quantification of levels of Cyclin E in MP2 lineage in 12 hpf control and pros mutant embryos using the plot-profile analysis of ImageJ. The plot profile is obtained within the boxed area and shown on the right. The arrowhead on the plot profile marks the expression profile for dMP2 in control and the undivided MP2-lineage cell in the mutant. The missing Odd-positive cell in the mutant (right panel) could be due to the direct differentiation of MP2 to vMP2 (an Odd-negative cell), or a staining artifact. (TIF) [file pgen.1010339.s004.tif]
